# Moving beyond information provision: strengthening structural and outcome-oriented approaches to food security among refugee populations

**DOI:** 10.1017/S136898002610295X

**Published:** 2026-06-24

**Authors:** Kervin Blanco Untalan, Genessa Jesy Teroy Pagote, Kyle Renz Gomez Tejero

**Affiliations:** 1 Food Technology, Bukidnon State University, Philippines; 2 Natural Science Department, Bukidnon State University, Philippines

**Keywords:** Refugee populations, Food security, Public health policy, Nutrition interventions

Dear Editor,

The recent article by Wood *et al.* ^(1)^ examines how Australian settlement workers use food security information resources with refugee clients and provides valuable insights into frontline reliance on translated, pictorial and simplified materials to address linguistic and literacy barriers. The study effectively highlights the practical challenges of delivering food-related information in culturally and linguistically diverse contexts, underscoring the importance of accessible communication strategies in supporting refugee populations.

These findings reinforce a well-established understanding that food security extends beyond access to information alone and is shaped by a complex interplay of structural, social and economic determinants. Evidence indicates that food insecurity among refugee and immigrant populations remains highly prevalent and is strongly associated with income instability, limited access to culturally appropriate foods and social exclusion^(2–4)^. While settlement workers serve as critical intermediaries in navigating food environments, reliance on resource-based approaches risks inadvertently shifting responsibility towards individuals and service providers rather than addressing broader systemic constraints.

Emerging evidence further suggests that although interventions such as nutrition education, food assistance and cash-based support are widely implemented, their long-term effectiveness remains inconsistent. A systematic review reported that while several interventions demonstrate short-term improvements in dietary outcomes, evidence of sustained food security impact on remains limited across refugee populations^(5)^. Structural barriers, including affordability, availability of culturally appropriate foods and transportation constraints, continue to restrict access to healthy food options, thereby limiting the effectiveness of information-based strategies^6^. In addition, research indicates that individuals frequently rely on heuristic cues, such as perceived familiarity or naturalness, when interpreting food-related information^(6)^, which may further constrain the translation of knowledge into sustained behavioural change.

Taken together, these findings suggest that information provision, while necessary, is insufficient in isolation. Future efforts would benefit from shifting towards outcome-oriented approaches that assess the extent to which interventions lead to measurable improvements in dietary practices and food security status. Interventions that incorporate co-design with refugee communities, alongside structural and policy-level support, have demonstrated greater relevance and sustainability^(5)^. Furthermore, strengthening policy frameworks that address underlying determinants of food insecurity, including economic access, social protection and inclusion within local food systems, may offer more durable and equitable solutions^(2)^.

In this context, the study by Wood *et al.* ^(1)^ provides an important foundation for understanding current practices in food security communication. Building on this work, there is a clear opportunity to advance more comprehensive, system-oriented strategies that integrate information provision with structural and policy interventions to more effectively support food security among refugee populations.
